# Assessment of Physical Health Investigations Completed Within 24 Hours of Admission in the Department of Psychiatry, Gulab Devi Teaching Hospital, Lahore

**DOI:** 10.1192/bjo.2026.11842

**Published:** 2026-06-30

**Authors:** Mudassar Ijaz, Syeda Areeba Siraj, Muhammad Mansoor Ali, Syed Ali Siraj, Ali Abbas Zahid

**Affiliations:** 1 Avicenna Hospital, Lahore, Pakistan; 2 Al Aleem Medical Collge / Gulab Devi Teaching Hospital, Lahore, Pakistan; 3 Rawalpindi Medical University, Rawalpindi, Pakistan

## Abstract

**Aims:**

To assess adherence to recommended baseline physical, laboratory, and additional investigations within 24 hours of admission in psychiatric inpatients, identify gaps, implement targeted interventions, and evaluate improvement in compliance.

ET AL AUTHORS :

6. Dr. Muhammad Zubair - General Practitioner- muhammadzubair1103@gmail.com

**Methods:**

A closed-loop quality improvement audit with a repeated cross-sectional design was conducted in the Psychiatry Department over 2 months. Fifty patients were included: 25 pre-intervention and 25 post-intervention phases. Pre-intervention data were collected retrospectively from patient records for physical examination (general + systemic), vital signs, BMI/weight, ECG, standard laboratory tests (CBC, LFTs, RFTs, serum electrolytes, fasting lipid profile, fasting glucose, viral markers), and special/indicated investigations (pregnancy test, urine drug screen, TFTs).

Documentation of findings in clinical notes was also assessed. Interventions included staff education sessions, standardized admission checklists, reminders during ward rounds, and coordination with laboratory and ECG services. Post-intervention data were collected prospectively using identical criteria to measure improvement.

**Results:**

Pre-intervention (n=25): Physical examination was documented in 72%, vital signs in 80%, ECG in 52%, and BMI/weight in 60%. Laboratory investigations showed completion rates of80% for CBC and LFTs/RFTs, 84% for serum electrolytes, 68% for fasting lipid profile and glucose, and 72% for viral markers. Pregnancy testing was completed in 7.7% of eligible females, urine drug screening in 0% of indicated cases, thyroid function tests in 20%, and documentation of findings in 56%.

Post-intervention (n=25): Physical examination improved to 92% (+20%), vital signs to 96% (+16%), ECG to 80% (+28%), and BMI/weight to 88% (+28%). Completion of CBC and LFTs/RFTs improved to 96% each (+16%), serum electrolytes to 92% (+8%), fasting lipid profile and glucose to 84% (+16%), and viral markers to 88% (+16%). Pregnancy testing in eligible females increased to 85% (+77.3%), urine drug screening to 75%, thyroid function testing to 80% (+60%), and documentation of findings to 92% (+36%).

**Conclusion:**

Baseline compliance with physical health investigations within 24 hours of psychiatric admission was variable, with significant gaps in ECGs, metabolic screening, special investigations, and documentation. Following targeted interventions, substantial absolute improvements were observed across all domains, particularly in ECG performance, metabolic monitoring, pregnancy testing, thyroid function testing, and documentation quality. This audit demonstrates that structured educational and system-based interventions can significantly enhance physical healthcare delivery in psychiatric inpatient settings. Ongoing re-audit is recommended to sustain improvements and ensure patient safety.

